# Phytochemical richness, antioxidant activity, and molecular diversity in *Papaver* species from Iran’s western and central regions

**DOI:** 10.3389/fpls.2025.1635867

**Published:** 2025-08-18

**Authors:** Naser Nazari, Fariba Sharifnia, Fahimeh Salimpour, Mohammad Mehrnia, Afsaneh Gran, Mehrdad Zarafshar

**Affiliations:** ^1^ Department of Biology, NT. C., Islamic Azad University, Tehran, Iran; ^2^ Lorestan Agricultural and Natural Resources Research and Education Center, AREEO, Khorramabad, Iran; ^3^ Department of Applied Botany, Federal University of Lavras (UFLA), MG, Brazil; ^4^ Linnaeus University, Faculty of Technology, Department of Forestry and Wood Technology, Växjö, Sweden

**Keywords:** *Papaver*, antioxidant activity, flavonoids, total phenols, molecular analysis

## Abstract

This study examined the phytochemical diversity, antioxidant capacity, and genetic relationships among *Papaver* species collected from western and central Iran. Significant interspecific and regional variation was observed in total phenolic and flavonoid contents, with *P. hybridum* from Khuzestan showing the highest phenolic (50.26 mg GAE/g DW) and antioxidant activity (DPPH: 70.40%, FRAP: 6.91 µmol Fe²^+^/g DW). *P. macrostomum* and *P. dubium* also exhibited notable flavonoid and anthocyanin levels, respectively. Codeine content peaked in *P. hybridum*, while papaverine was highest in *P. dubium*. Antioxidant traits correlated strongly with phenolic content and environmental variables such as humidity and elevation. PCA revealed clustering of antioxidant traits, while alkaloids and anthocyanins formed distinct patterns. Phylogenetic analysis indicated complex evolutionary relationships and possible introgression among species. These findings highlight substantial phytochemical and genetic variation shaped by local environments. As this study focuses on Iranian populations, broader geographic sampling is needed to generalize the patterns observed.

## Graphical Abstract

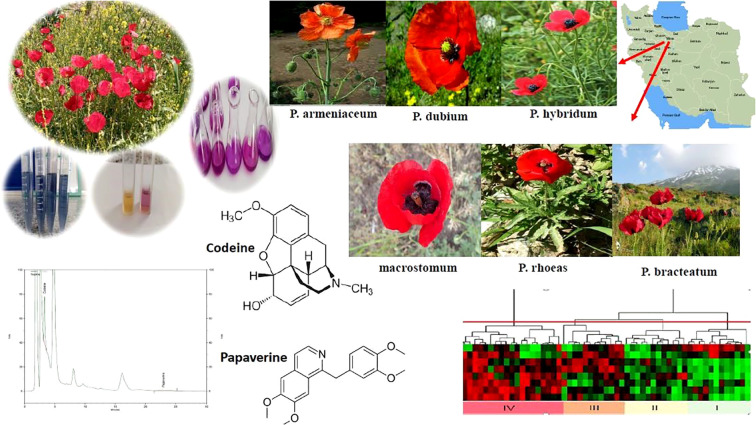

## 1 Introduction

Medicinal plants have long been recognized as reservoirs of bioactive compounds, shaping the foundation of both traditional and modern medicine (Guo et al., 2024). Among them, species within the genus *Papaver* (family Papaveraceae) stand out for their unique pharmacological attributes, particularly their role as primary sources of narcotic alkaloids such as thebaine, codeine, and morphine (Májer and Németh, 2024). These alkaloids, biosynthesized through complex metabolic pathways from L-tyrosine, have been extensively utilized in the pharmaceutical industry for pain management, muscle relaxation, and sedative applications (da Silva et al., 2025). Despite their significance, the genetic and phytochemical diversity of *Papaver* species remains insufficiently explored, especially in regions with high species variability, such as Iran.

Among these species, *P. bracteatum*, native to temperate regions of Iran, has gained attention for its potential pharmaceutical applications due to its high content of bioactive alkaloids (Shaghaghi et al., 2019). *P. rhoeas*, commonly known as the field poppy, is traditionally used for its mild sedative effects and antioxidant-rich petals, which contain flavonoids and anthocyanins with potential health benefits (Dif et al., 2015). However, despite their medicinal potential, limited research has focused on the molecular characterization and phytochemical profiling of *Papaver* species in Iran.

Environmental factors such as altitude, soil composition, and climate variations significantly influence alkaloid biosynthesis, leading to considerable intraspecific variability (da Silva et al., 2025). Understanding these variations through genetic and biochemical analyses is crucial for optimizing cultivation strategies, improving pharmaceutical applications, and ensuring the sustainable use of these valuable plant resources.

The Internal Transcribed Spacer (ITS) region of ribosomal DNA has emerged as a robust marker for phylogenetic analysis, offering insights into genetic diversity and taxonomic classifications (Lee et al., 2010). ITS-based studies have been instrumental in elucidating genetic relationships in diverse plant taxa, for instance, *Medicago sativa* (Furan et al., 2024). Given the increasing application of molecular markers in forensic botany and biotechnological research, genetic profiling of *Papaver* species holds significant promise for both pharmaceutical advancements and regulatory frameworks aimed at controlling illicit opium production.

Beyond genetics, phytochemical profiling is essential to understanding the metabolic plasticity of *Papaver* species. Alkaloid composition is known to fluctuate in response to environmental factors, developmental stages, and organ-specific metabolic activities. Dif et al. (2015) highlighted substantial variation in polyphenolic content across different tissues of *P. rhoeas*, with petals exhibiting the highest concentrations of bioactive compounds. Despite these findings, comprehensive molecular and phytochemical studies on *Papaver* species, particularly in Iran—a region known for its ecological diversity and endemic plant taxa—remain scarce. For instance, Nematolahi et al. (2024) investigated the morphological and phytochemical diversity among *Papaver bracteatum* populations in Iran. Their findings highlight significant morphological variations, suggesting that amino and fatty acids could serve as effective tools for infra-species classification. Qaderi et al. (2019) demonstrated significant genetic diversity and phytochemical variability just in *Papaver bracteatum* using SCoT and ISSR markers, identifying three major genetic groups linked to eco-geographical regions. Moreover, Salehi et al. (2007) analyzed four native *Papaver* species from Iran—*P. glaucum, P. tenuifolium, P. dubium*, and *P. fugax*—for their narcotic alkaloid content using HPLC. *P. dubium* and *P. glaucum* contained morphine, codeine, and thebaine, whereas *P. fugax* lacked morphine, and *P. tenuifolium* was free of codeine. This study provides valuable insights into the phytochemical composition of Iranian *Papaver* species.

This study aims to bridge this knowledge gap by conducting an integrated genetic and phytochemical investigation of six *Papaver* species (*P. dubium, P. hybridum, P. rhoeas, P. armeniacum, P. macrostomum, P. bracteatum*) collected from western and central Iran (From four provinces. Using ITS markers, we seek to unravel phylogenetic relationships and species differentiation, while simultaneously quantifying key alkaloids, including papaverine and codeine, to assess their biosynthetic potential. By combining molecular and phytochemical approaches, this research will contribute valuable insights into the evolutionary dynamics, biochemical pathways, and conservation strategies for *Papaver* species, with broader implications for medicinal plant research and pharmaceutical applications.

## 2 Materials and methods

### 2.1 Plant material

This study, conducted in growing season of 2019, involved the collection of *Papaver* spp. samples from various species across the western and central regions of Iran, including Kermanshah, Tehran, Khuzestan, and Lorestan (**Table 1**).

**Table 1:** List of *Papaver* species and their collection areas in Iran.

No	Location	Species	Climate
1	Khuzestan, Behbahan Area,	*P. dubium*	Elevation (200–1500 m.s.l), Mean Precipitation (280 mm), Relative humidity (32%), T_max_ and T_min_ (51 and 5 centigrade)
2	Khuzestan, Andimeshk Area	*P. hybridum*	
3	Khuzestan, Andimeshk Area, Pole’zal,	*P. macrostomum*	
4	Khuzestan, Andimeshk, Daz Dam,	*P. rhoeas*	
5	Lorestan, Dorod Area,Gahar Lake,	*P. armeniacum*	Elevation (1400–2700 m.s.l), Mean Precipitation (640 mm), Relative humidity (53%), T_max_ and T_min_ (47 and -8 centigrade)
6	Lorestan, Aligodarz Area, Oshtorankoh,	*P. dubium*	
7	Lorestan, Dorod Area, Bishe Village	*P. macrostomum*	
8	Tehran, Damavand Area, Amamzade Hashem	*P. armeniacum*	Elevation (900–1800 m.s.l), Mean Precipitation (475 mm), Relative humidity (46%), T_max_ and T_min_ (43 and -15 centigrade)
9	Tehran, Ploor Area, Haraz Road,	*P. bracteatum*	
10	Tehran, Gagrood Area,	*P. dubium*	
11	Tehran, Firozkoh Area,	*P. hybridum*	
12	Tehran, Latian Dam	*P. macrostomum*	
13	Kermanshah, Kamyaran Area, Vazmale Village,	*P. armeniacum*	Elevation (1350–2500 m.s.l), Mean Precipitation (615 mm), Relative humidity (53%), T_max_ and T_min_ (43 and -12 centigrade)
14	Kermanshah, Ghasrshirin Area	*P. hybridum*	
15	Kermanshah, Azad University	*P. macrostomum*	

For genetic studies, fresh plant material was collected, identified, and preserved under controlled temperature and light conditions in an herbarium. The samples were then transferred to the laboratory, where DNA extraction was carried out following standard protocols. To assess phytochemical composition, a separate portion of each sample was immediately air-dried under shade at room temperature to minimize compound degradation. The dried samples were stored in airtight containers at -20°C until further chemical analysis. This ensured the stability of key metabolites, including alkaloids, flavonoids, and phenolics.

### 2.2 Extract preparation

Ten grams of *Papaver* leaves were oven-dried at 70°C, followed by extraction in 100 mL of 85% methanol for 48 hours at 25°C. The extracts were then filtered, evaporated under reduced pressure, and freeze-dried. Finally, the samples were stored at -80°C until analysis for antioxidant activity.

### 2.3 Total phenol content

The total phenol content was determined using the Folin-Ciocalteu method. First, 125 µL of 10% Folin reagent was added to the extracted methanolic sample, followed by incubation at 35°C for 5 minutes. Subsequently, 200 µL of 7% sodium bicarbonate solution was added, and the mixture was homogenized. Absorbance was measured at 765 nm using a UV-3200 spectrophotometer (MAPADA, Shanghai Mapada Instruments Co., Ltd., China). Results were expressed as mg of gallic acid per gram of dry tissue (Meyers et al., 2003).

### 2.4 Total flavonoid content

The total flavonoid content (TFC) was determined using the aluminum chloride colorimetric method. First, 1.7 mL of 30% ethanol, 150 µL of 0.5 mM sodium nitrite, and 150 µL of 0.3 mM aluminum chloride were added to 150 µL of methanolic extract and homogenized. After 5 minutes, 100 µL of 1 mM sodium hydroxide solution was added. The absorbance was then measured at 510 nm using a UV-3200 spectrophotometer (MAPADA, Shanghai Mapada Instruments Co., Ltd., China). The total flavonoid content was expressed as mg quercetin equivalent (QUE) per gram of dry weight (DW) (Du et al., 2009).

### 2.5 Antioxidant activity

The antioxidant activity of the plant was assessed using the DPPH (2,2-diphenyl-1-picrylhydrazyl) free radical scavenging assay, following the spectrophotometric method described by Brand-Williams et al. (1995). For this, 800 µL of DPPH solution (0.004 g/100 mL of 80% methanol) was added to 200 µL of methanolic extract, and the mixture was incubated in the dark for 30 minutes. The absorbance was then measured at 517 nm using a UV-3200 spectrophotometer (MAPADA, Shanghai Mapada, China). The antioxidant capacity of the extracts was expressed as DPPH inhibition percentage, calculated using the following formula:


$$\text{DPPH}\%=(\frac{A1-A2}{A1})\times 100$$

where A_1_ is the absorbance of the control (DPPH solution without extract), and A_2_ is the absorbance of the sample.

Antioxidants reduce Fe³^+^ to Fe²^+^, forming a blue TPTZ–Fe²^+^ complex detected at 593 nm. For the FRAP assay, 250 µg/mL plant extract was added to 2 mL FRAP reagent (10 mM TPTZ in 40 mM HCl, 20 mM FeCl_3_, 300 mM acetate buffer, pH 3.6), incubated at 37 °C for 10 min, and absorbance measured at 593 nm. A FeSO_4_·7H_2_O standard curve (125–1000 µM) was used to express antioxidant capacity as µmol Fe²^+^/g fresh weight (Benzie and Strain, 1996).

### 2.6 Total anthocyanin content

To quantify anthocyanin content, 1 g of dried petals was powdered and extracted using 10 mL of acidified methanol (1% HCl, v/v). The mixture was sonicated for 30 min at 20 ± 2°C, followed by storage in the dark at 4°C for 12 h with gentle stirring. After centrifugation at 10,000 rpm for 15 min, the supernatant was collected and stored at -20°C until further analysis.

Total anthocyanin content was measured using the modified pH differential method (Virgen-Ortiz et al., 2020). A portion of the extract was diluted (1:10) with 0.025 mol L^−^¹ potassium chloride buffer (pH 1.0) and 0.4 mol L^−^¹ sodium acetate buffer (pH 4.5). The absorbance was recorded at 510 nm and 700 nm at both pH levels using a UV-3200 spectrophotometer (MAPADA, Shanghai Mapada, China). The anthocyanin content was calculated using the following equation (Virgen-Ortiz et al., 2020).


$$\begin{array}{c} \text{Anthocyanins}=[({\text{A}}_{510}\text{nm}-{\text{A}}_{700}\text{nm}{)}_{pH\;1.0}-({\text{A}}_{510}\text{nm}\\ -{\text{A}}_{700}\text{nm}{)}_{pH\;4.5}]\times \text{MW}\times \text{V}\times \text{D}\times 1000/\epsilon \times \text{L}\\ \times \text{w} \end{array}$$

Where A510 - A700 represents the absorbance difference at 510 nm and 700 nm for pH 1.0 and pH 4.5. The MW refers to the molecular weight of cyanidin-3-glucoside (449.2 g/mol), V is the final volume of the extract in milliliters, DF is the dilution factor, ϵ is the molar extinction coefficient of cyanidin-3-glucoside (26,900 L/mol·cm), L is the path length of the cuvette in centimeters, and W is the sample weight in grams. This formula allows for the calculation of the total anthocyanin content in the sample. Finally, the anthocyanin content is quantified as mg of pelargonidin-3-glucoside per 100 gr of dry weight.

### 2.7 Alkaloid content analysis

The poppy petal samples were ground using a laboratory grinder (IKA, Staufen, Germany) and sieved through a 0.5 mm mesh. The resulting powdered material was then extracted to quantify the codeine and papaverine, via High-Performance Liquid Chromatography (HPLC, Agilent 1290 Infinity II). For analysis, standard solutions with concentrations of 15, 25, and 50 mg/L for both alkaloids were prepared and injected into the HPLC system. Calibration curves for codeine and papaverine were generated by plotting the area under the corresponding peaks as a function of concentration. To prepare the sample extracts, 50 mg of dry powder was mixed with 1 mL of methanol (HPLC-grade) and placed in an ultrasonic bath for 15 minutes to facilitate the dissolution of alkaloid compounds. The resulting mixture was filtered through a 0.45 µm filter before being injected into the HPLC system. The alkaloid concentrations of codeine and papaverine in the sample solutions were determined based on the area under the respective peaks and the corresponding calibration curves, with data analysis performed using the system’s software. Finally, the alkaloid content in the dry extracts was calculated based on the amount of dry material used to prepare the sample solution.

### 2.8 Isolation of total DNA

Dried leaves were treated with 70% ethyl alcohol to prevent fungal contamination prior to genomic DNA isolation (Doyle & Doyle, 1987). The samples were homogenized using a pestle, and genomic DNA was extracted from the powdered material using the DNeasy Plant Mini Kit (Qiagen, Germany). The quality and quantity of the isolated DNA were assessed via 1.5% agarose gel electrophoresis. The extracted DNA was then stored at -20°C for future use.

### 2.9 Molecular markers and PCR amplification

PCR amplification was carried out in a total reaction volume of 50 µL, containing 5 µL of Gold 10X buffer (500 mM KCl, 100 mM Tris–HCl pH = 8.3, 15 mM MgCl2, 1% Triton X-100, 1600 µg/mL BSA, 2 mM dNTP pH = 8.2–8.4), 20 pmol of forward and reverse primers, 2.5 units of AmpliTaq Gold™ DNA polymerase, sterile distilled water, and 10 ng of genomic DNA. The PCR conditions included an initial denaturation step at 95°C for 11 minutes, followed by 28 cycles of 1 minute at 95°C, 1 minute at 50°C, and 1 minute at 72°C, with a final extension at 72°C for 7 minutes. The amplification was performed using an ABI 9700 thermal cycler (Applied BioRad, USA). The ITS1 and ITS4 primer pairs were used with sequences 5’-TCCGTAGGTGAACCTGCGG-3’ and 5’-TCCTCCGCCTTATTGATATGC-3’ (Hsiao et al., 1994), respectively. PCR products were analyzed using 2% agarose gel electrophoresis for qualitative assessment.

The location of DNA fragments in the gel was determined by staining with ethidium bromide. The PCR product was loaded into the well, and the DNA migration rate was directly proportional to the applied voltage. To estimate fragment sizes, a 50-bp DNA ladder was used as a marker. After electrophoresis, the DNA fragments of interest were excised from the gel, purified, and sent for sequencing. Once the FASTA sequence file was received, the sequences were edited using the corresponding chromatogram file and then subjected to a BLASTN search. The closest matching sequences retrieved from the NCBI database were aligned and used to construct a phylogenetic tree.

### 2.10 Statistical analysis

Because not all species were collected across all provinces, the dataset does not constitute a fully crossed factorial design. Accordingly, each site-species combination (n=15) was treated as an independent treatment in a one-way ANOVA model. This approach allowed us to compare the means of these combinations without assuming complete species × region interactions. While LSD was used initially for pairwise comparisons, Tukey’s HSD alternatively had applied to control family-wise error in unbalanced designs. Data analysis was conducted using SAS software (PROC GLM, v. 9.4). Moreover, a Mantel test was performed to assess the correlation between genetic distance (based on molecular markers) and biochemical distance (based on phytochemical profiles) among populations. Multivariate analyses, including principal component analysis (PCA) and heat map correlation (HMC), were carried out using GraphPad Prism (v. 10.4.1). Kinship relationships were assessed using MEGA7 software, and neighbor-joining phylograms were constructed in MEGA.

## 3 Results

### 3.1 Secondary metabolite analysis in *Papaver* species

**Figure 1:**
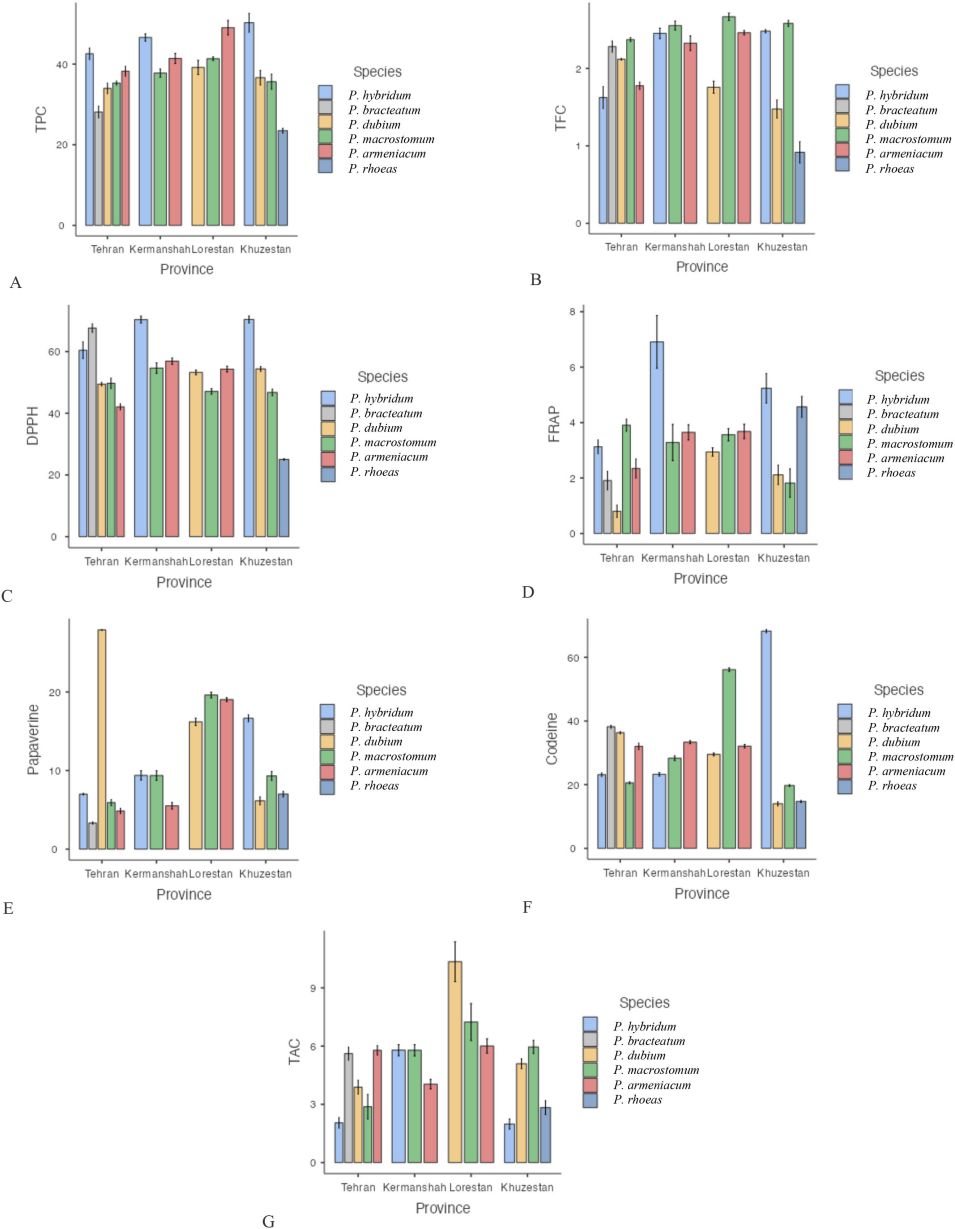
Mean comparison of phytochemical traits in species of the genus *Papaver* collected from different regions. More explanation: **(A)** (TPC (mg GAE/g DW)= total phenolic content), **(B)** (TFC (mg QUE/g DW)=total flavonoid content), **(C)** (DPPH (%)=antioxidant activity), **(D)** (FRAP (µmol Fe²^+^ g^−^¹ DW)= ferric reducing antioxidant power, **(E)** (Papaverine (mg/g DW), alkaloid), **(F)** (Codeine (mg/g DW)= alkaloid) and **(G)** (TAC (μmol/g DW)= total anthocyanin content).

**Figure 2:**
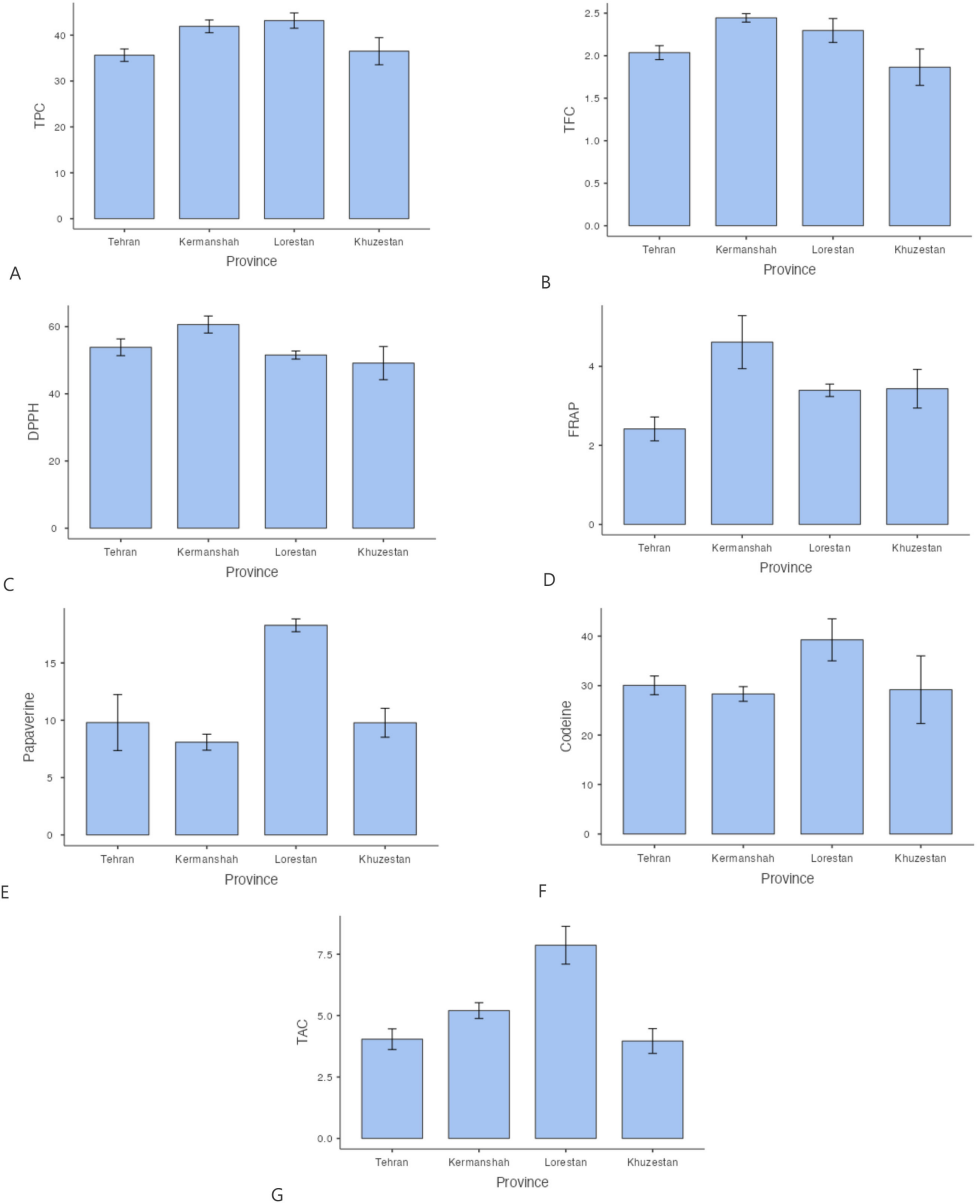
Mean comparison of phytochemical traits in Papaver between different regions. More explanation: **(A)** (TPC (mg GAE/g DW)= total phenolic content), **(B)** (TFC (mg QUE/g DW)=total flavonoid content), **(C)** (DPPH (%)=antioxidant activity), **(D)** (FRAP (µmol Fe²^+^ g^−^¹ DW)= ferric reducing antioxidant power, **(E)** (Papaverine (mg/g DW), alkaloid), **(F)** (Codeine (mg/g DW)= alkaloid) and **(G)** (TAC (μmol/g DW)= total anthocyanin content).

**Figure 3:**
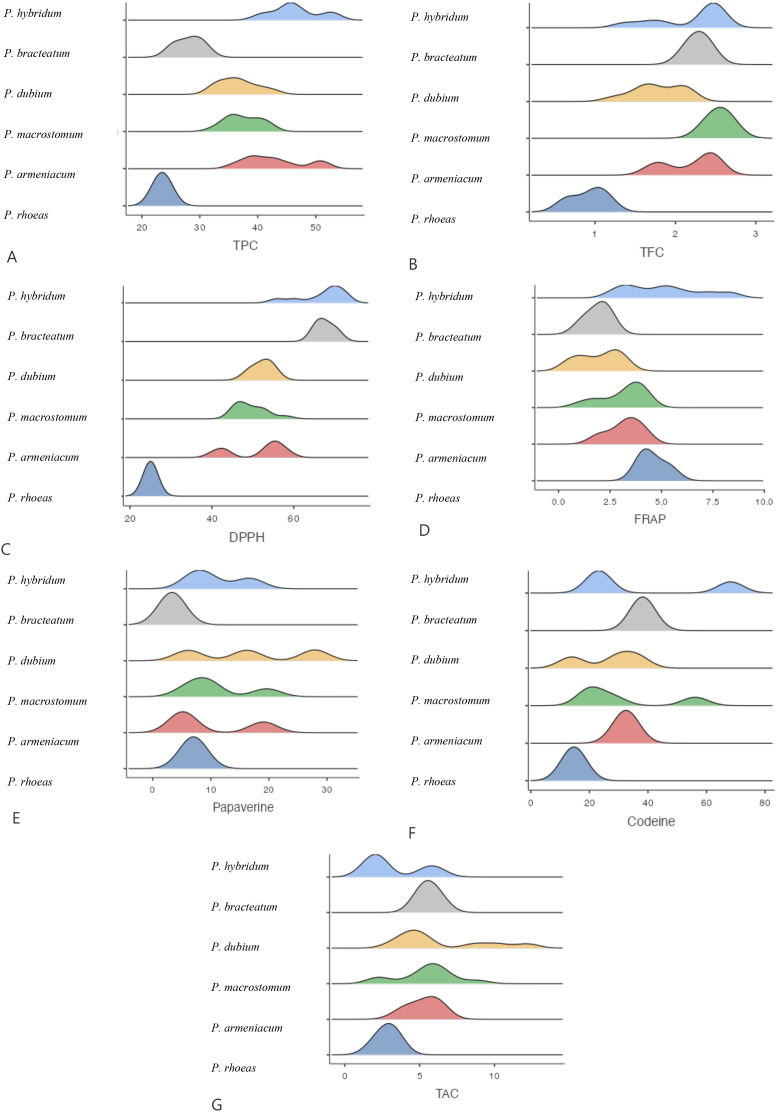
Mean comparison of phytochemical traits in different Papaver. More explanation: **(A)** (TPC (mg GAE/g DW)= total phenolic content), **(B)** (TFC (mg QUE/g DW)=total flavonoid content), **(C)** (DPPH (%)=antioxidant activity), **(D)** (FRAP (µmol Fe²^+^ g^−^¹ DW)= ferric reducing antioxidant power, **(E)** (Papaverine (mg/g DW), alkaloid), **(F)** (Codeine (mg/g DW)= alkaloid) and **(G)** (TAC (μmol/g DW)= total anthocyanin content).

Our analysis of total phenolic and flavonoid content across different *Papaver* species and geographic regions revealed significant variations. The results indicated that *P. hybridum* exhibited the highest total phenol content among the studied species, particularly in the Khuzestan region (50.26 mg GAE/g DW), whereas *P. rhoeas* had the lowest phenolic content (23.5 mg GAE/g DW in Khuzestan) (**Figure 1**). For total flavonoid content, *P. macrostomum* exhibited the highest values across multiple regions, including Lorestan (2.66 mg QUE/g DW), Khuzestan (2.58 mg QUE/g DW), and Kermanshah (2.55 mg QUE/g DW). The lowest flavonoid content was recorded in *P. rhoeas* from Khuzestan (0.91 mg QUE/g DW). Overall, *Papaver* samples from Lorestan and Kermanshah exhibited higher levels of both phenolic and flavonoid compounds (**Figure 2**). When analyzing species independent of region, *P. hybridum* consistently showed the highest phenolic and flavonoid content, while *P. rhoeas* contained the lowest levels across all tested populations (**Figure 3**).

The antioxidant activity of *Papaver* species was assessed using both DPPH and FRAP assays. The highest DPPH radical scavenging activity was recorded in *P. hybridum* from Khuzestan and Kermanshah provinces, with values of 70.40% and 70.33%, respectively (**Figure 1**). In contrast, the lowest DPPH activity was observed in *P. rhoeas* from Khuzestan (25.03%). Similarly, the results obtained from the FRAP assay indicated that *P. hybridum* from Kermanshah province exhibited the highest ferric reducing antioxidant power (6.91 µmol Fe²^+^ g^−^¹ DW), whereas the lowest FRAP activity was found in *P. dubium* from Tehran (0.799 µmol Fe²^+^ g^−^¹ DW) (**Figure 1**). Overall, *P. hybridum*, which exhibited the highest levels of both phenolic and flavonoid compounds, also demonstrated the strongest antioxidant activity in both assays. The similarity in ranking between these two methods suggests that antioxidant mechanisms in *P. hybridum* involve both radical scavenging and reducing activities. Interestingly, *P. rhoeas* from Khuzestan, which had the lowest total phenolic and flavonoid content, also exhibited the lowest DPPH scavenging activity. Overall, *Papaver* samples Kermanshah exhibited higher levels of both DPPH and FRAP (**Figure 2**). Moreover, *P. hybridum* demonstrated the highest DPPH and FRAP concentrations (**Figure 3**).

As shown in **Figure 1**, codeine content was highest in *P. hybridum* from Khuzestan (68.25 mg/g DW), while *P. dubium* from the same region exhibited the lowest level (14.00 mg/g DW). In contrast, papaverine was most abundant in *P. dubium* from Tehran (27.90 mg/g DW) and least abundant in *P. bracteatum* from the same province (3.32 mg/g DW). The plant samples of Papaver from Lorestan showed the greatest level of codeine and papaverine (**Figure 2**). Overall, *P. hybridum* demonstrated the highest codeine concentration, whereas *P. dubium* from Lorestan contained the highest papaverine levels, indicating significant interspecific and regional variation in alkaloid biosynthesis (**Figure 3**).

Anthocyanins are a subclass of flavonoids responsible for pigmentation in plants and have well-documented antioxidant properties. Among the studied samples, *P. dubium* from Lorestan exhibited the highest total anthocyanin content (**Figure 1**). The plant samples from Lorestan province exhibited the higher concentration of TAC. (**Figure 2**) The remaining *Papaver* species did not exhibit significant differences in anthocyanin levels (**Figure 3**).

**Figure 4:**
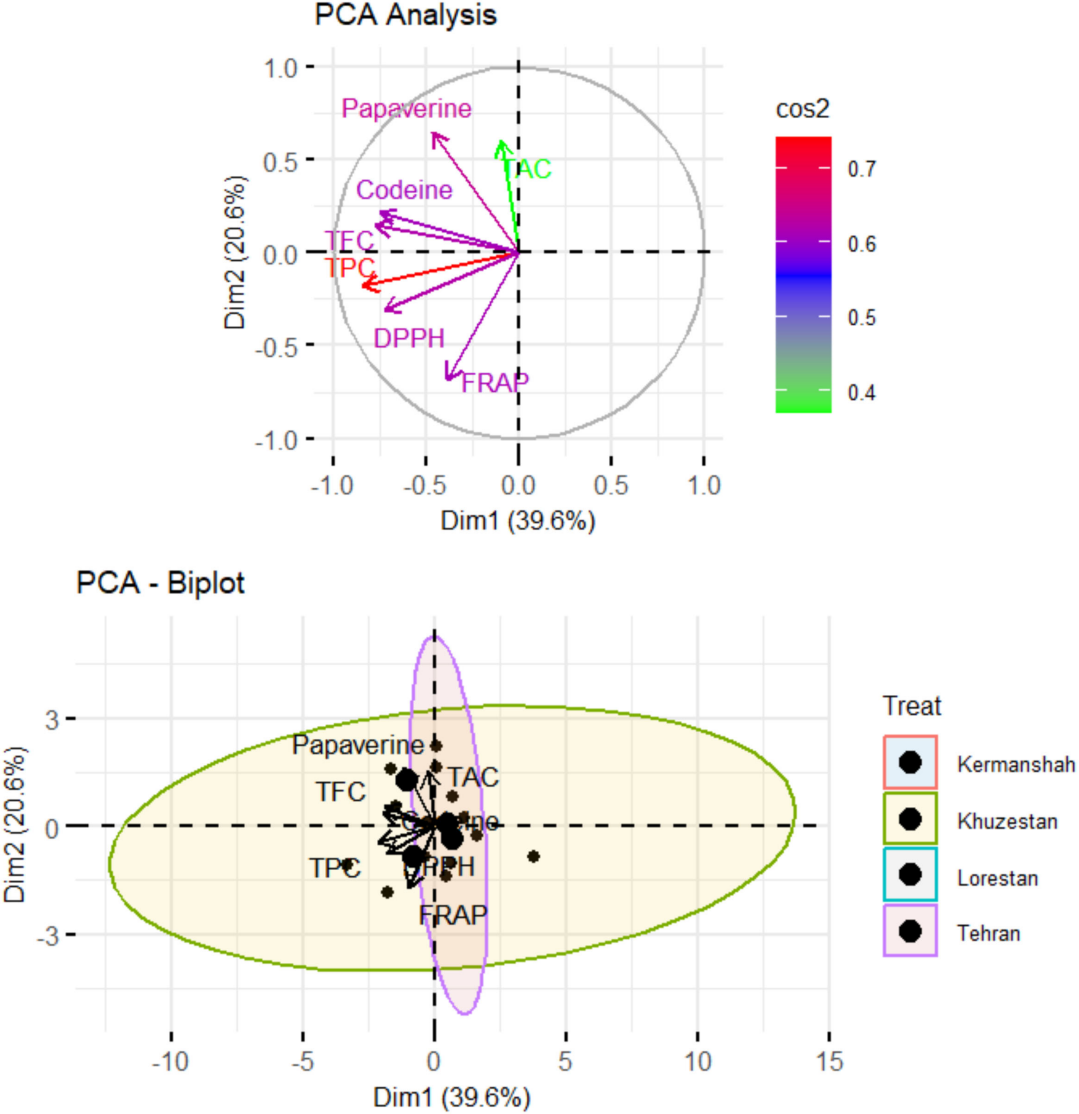
PCA biplot showing sample distribution by treatment and biochemical traits. More explanation: PCA biplot of *Papaver* biochemical traits (Above). Arrows show each variable’s contribution and correlation with Dim1 (39.6%) and Dim2 (20.6%). The color gradient (cos²) indicates representation quality, with red denoting stronger contributions. In the bottom bioplat, the studied sites are scattered with respect to the biochemical variables.

The PCA analysis (**Figure 4**) revealed distinct clustering patterns among the biochemical compounds measured in *Papaver* species, with the first principal component (Dim1) accounting for 39.6% of the total variance and the second principal component (Dim2) explaining 20.6%. Antioxidant-related parameters, including DPPH, FRAP, total phenolic content (TPC) were closely grouped, indicating a strong positive correlation among them. In contrast, alkaloids such as codeine and papaverine were positioned separately, suggesting their biosynthesis follows an independent pathway. Total anthocyanin content (TAC) was distinctly oriented along Dim2, indicating its contribution to a different variance structure than the other measured compounds.

When the provinces were scattered based on the studied variables, the samples from Khuzestan showed a wider spread along Dim1, reflecting higher variability in their phytochemical and antioxidant profiles, while the other regions, especially Tehran, exhibited tighter clustering, indicating more similar compositions. Overall, no clear regional separation was observed in the PCA biplot, suggesting a generally high chemical similarity among the populations, with most of the variation primarily driven by Dim1.

**Figure 5:**
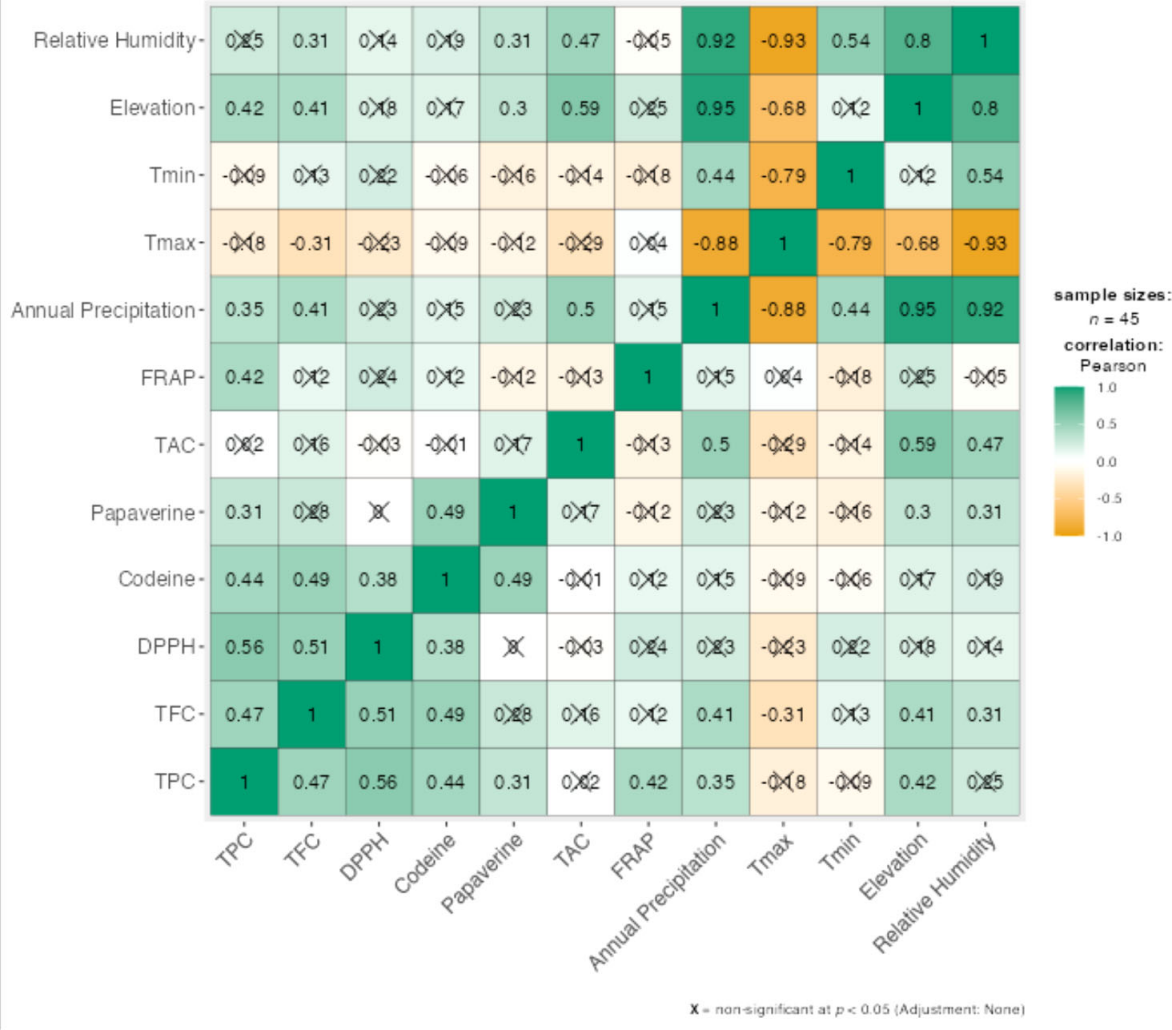
Correlations between biochemical traits and climatic variables.

The correlations between all biochemical variables and selected climatic parameters are shown in the **Figure 5**. Relative humidity, elevation, and annual precipitation consistently exhibited positive and significant correlations with most biochemical variables, whereas mean maximum temperature (Tmax) and mean minimum temperature (Tmin) generally showed weak and mostly non-significant correlations. Among the biochemical traits, total anthocyanin content demonstrated the strongest and most significant correlations with the climatic variables.

### 3.2 Molecular analysis

A phylogenetic tree was constructed using the Maximum Likelihood (ML) method with a bootstrap value of 500 and a Gamma Distribution parameter (G = 5), implemented in MEGA5 software. The analysis was based on gene sequences from various *Papaver* species.

**Figure 6:**
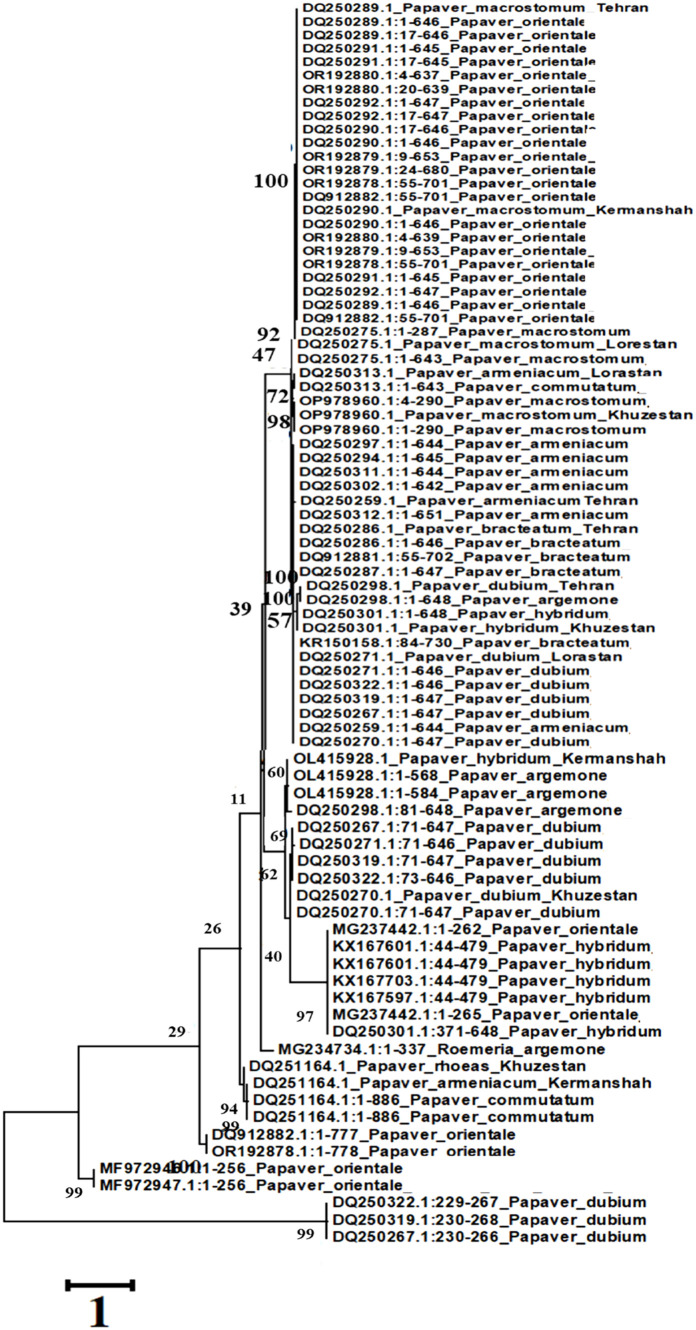
Phylogeny tree resulting from ITS analysis.

The resulting phylogeny (**Figure 6**) revealed a close evolutionary relationship among *Papaver orientale*, *P. macrostomum*, *P. bracteatum*, and some accessions of *P. armeniacum* and *P. dubium*, all clustering within a major clade. Notably, three sequences from *P. argemone* formed a distinct subgroup with other accessions of *P. dubium*, indicating genetic affinity between these species. A few divergent sequences from *P. dubium* and *P. orientale* appeared in separate, isolated clades, suggesting intraspecific genetic variability or possible introgression events.

Interestingly, all *P. hybridum* sequences grouped together and were also associated with two sequences from *P. orientale*, suggesting a shared genetic background or gene flow between these taxa. The lack of monophyly in the *Papaver* and *Rhoeadium* clades, as inferred from the maximum parsimony analysis, further supports the hypothesis that current taxonomic classifications may not fully reflect the evolutionary history of these genera.

A subset of sequences from *P. commutatum* and *P. rhoeas* also formed a separate group, reinforcing their distinction within the genus. These findings underscore the complex evolutionary history and taxonomic challenges within *Papaver* and related genera.

### 3.3 Genetic–biochemical distance correlation

**Figure 7:**
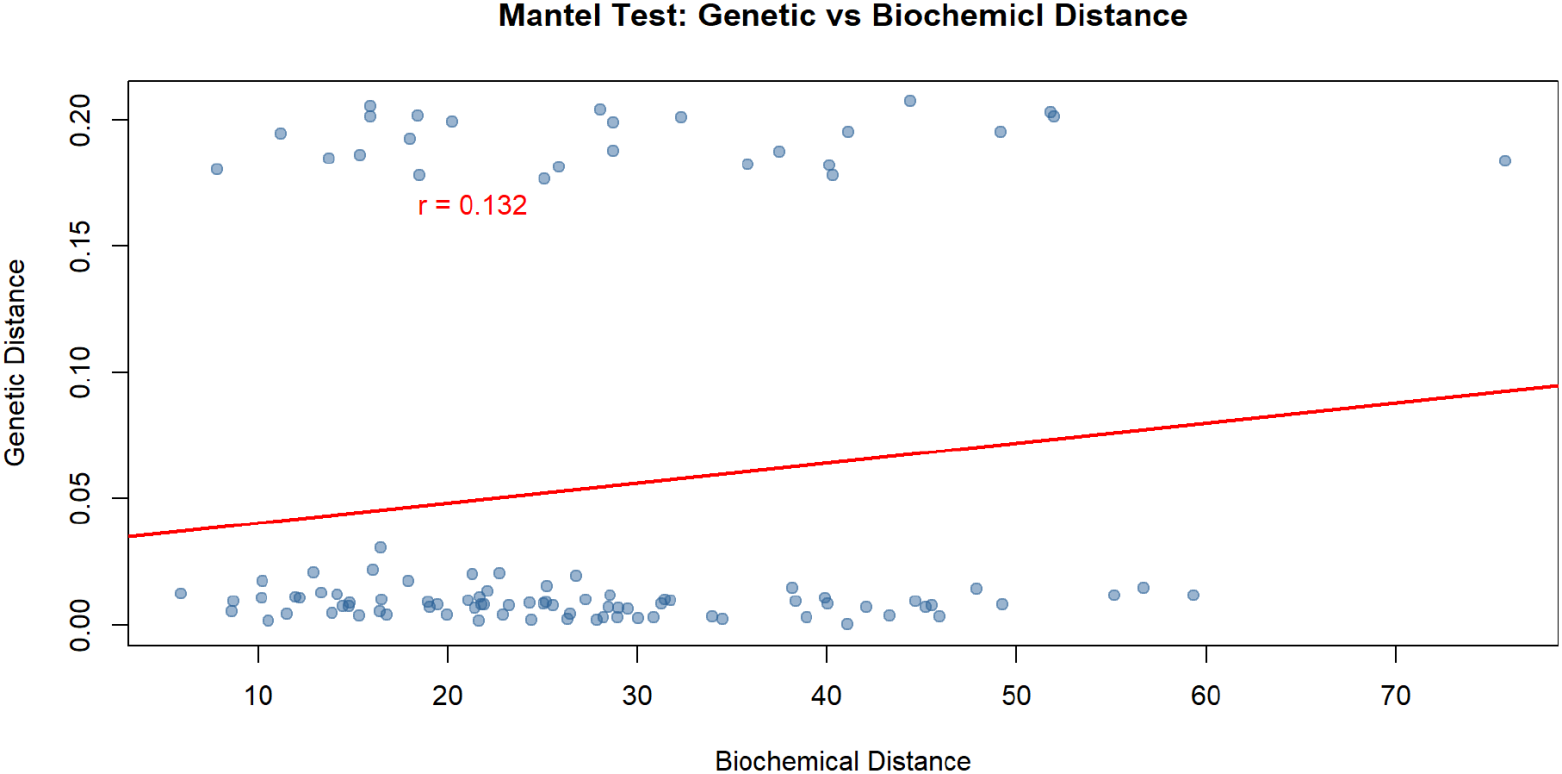
Mantel test showing the correlation between genetic distance and biochemical dissimilarity among Papaver populations.

The Mantel test revealed a weak positive correlation between genetic and biochemical distances among the studied populations (r = 0.132) (**Figure 7**). This low correlation coefficient indicates that there is only a limited association between the patterns of genetic variability and the phytochemical composition of the samples. In other words, differences in genetic makeup do not strongly predict differences in biochemical profiles across populations. This suggests that factors beyond genetic variation—such as environmental conditions, phenotypic plasticity, or local adaptation—may have a considerable influence on the observed chemical diversity.

## 4 Discussion

Secondary metabolites play a crucial role in plant defense mechanisms, ecological interactions, and pharmacological applications (Panahirad et al., 2023). Among these, phenolic compounds and flavonoids are widely recognized for their antioxidant, antimicrobial, and therapeutic properties (Tungmunnithum et al., 2018). These differences may be attributed to species-specific metabolic pathways, environmental stress factors, and genetic regulation of phenolic biosynthesis (Zhan et al., 2022). The observed regional differences may be linked to variations in soil composition, climatic conditions, and altitude, which are known to influence flavonoid biosynthesis (Alami et al., 2024).

Totally, the higher levels of secondary metabolites and antioxidant capacity were observed in the plant samples from Lorestan and Kermanshah, where elevation and annual precipitation are higher than in the other provinces. This data illustrates that environmental factors such as altitude and moisture availability can play a crucial role in regulating the biosynthesis of phenolic compounds and antioxidant enzymes (Chaves et al., 2009). Elevated habitats often expose plants to increased UV radiation, temperature fluctuations, and other abiotic stresses that stimulate the production of protective secondary metabolites (Hasanuzzaman et al., 2020). Moreover, higher precipitation can support better nutrient uptake and metabolic activity, further enhancing the accumulation of bioactive compounds (Sampaio et al., 2016).

The antioxidant activity of plant extracts is largely influenced by their phenolic and flavonoid content, which serve as potent free radical scavengers (Kähkönen et al., 1999). The DPPH assay primarily measures the ability of antioxidants to donate hydrogen atoms to neutralize free radicals, while the FRAP method evaluates the reducing power of the extracts by measuring Fe³^+^-TPTZ reduction to Fe²^+^-TPTZ (Munteanu and Apetrei, 2021). This aligns with studies that report a lower antioxidant potential in species with lower polyphenolic composition (Cai et al., 2004; Wojdyło et al., 2007). The regional differences observed in antioxidant activity may be attributed to environmental factors such as climate, soil composition, and altitude, which can significantly influence the biosynthesis of secondary metabolites (Jaakola and Hohtola, 2010). Our investigation clearly showed that climatic factors such as elevation and precipitation have a substantial impact on antioxidant activity, as evidenced by the consistently higher levels observed in samples from Kermanshah Province.

Alkaloids, comprising over 12,000 distinct chemical structures, represent one of the most diverse and significant groups of secondary metabolites (Khan et al., 2013). Alkaloids play a crucial role in plant defense mechanisms, primarily as chemical deterrents against herbivores and pathogens (Facchini, 2001). Codeine and papaverine are key benzylisoquinoline alkaloids (BIAs) with significant pharmacological applications. Codeine is widely used as an analgesic and cough suppressant, while papaverine functions as a vasodilator (Desgagné-Penix, 2021). The variation in their concentrations across different species and regions suggests species-specific metabolic pathways that prioritize the synthesis of particular alkaloids. Studies have shown that alkaloid content in *Papaver* species is influenced by environmental stressors such as temperature fluctuations, drought, and soil nutrients, which modulate gene expression in alkaloid biosynthetic enzymes (Desgagné-Penix et al., 2012). Additionally, previous research has reported that *Papaver* species exhibit distinct alkaloid profiles based on their genetic background. For example, Shaghaghi et al. (2019) found significant differences in alkaloid accumulation among various *Papaver* species, supporting the idea that both genetic and environmental factors drive these variations. Our results align with these findings, reinforcing the importance of considering both intrinsic and extrinsic factors in alkaloid research. These patterns did not correspond clearly with geographic origin, as samples from Tehran, Lorestan, Kermanshah, and Khuzestan provinces were distributed across several clades. This inconsistency may reflect environmental heterogeneity, latitudinal variation, or anthropogenic influences such as selective breeding and cultivation practices over time, which can impact genetic structure (Chai et al., 2024; Victory et al., 2006; Zhou et al., 2018).

Previous literature supports the observed relationships. For instance, Kadereit (1996) identified *P. apulum*, *P. argemone*, *P. hybridum*, and *P. pavonium* as closely related semi-rosette species distributed across southeastern Europe and into Asia. Carolan et al. (2006) provided molecular evidence that diploid *P. bracteatum* contributes to the genome of hexaploid *P. pseudo-orientale*, and that *P. commutatum*, *P. dubium*, and *P. rhoeas* are part of the *Rhoeadium* subgenus, with a broad but disjunct distribution across Eurasia.

A subset of sequences from *P. commutatum* and *P. rhoeas* also formed a separate group, reinforcing their distinction within the genus. Moreover, phylogenomic studies have indicated that *P. somniferum*, *P. rhoeas*, and *P. orientale* form a closely related trio within the Papaveraceae, clustering with *Coreanomecon hylomeconoides* (Zhou et al., 2018). Broader molecular systematics suggest that the order Ranunculales, which includes Papaveraceae, shares evolutionary roots with families like Lardizabalaceae (*Akebia quinata*, *Decaisnea insignis*) and Circaeasteraceae (*Kingdonia uniflora*, *Circaeaster agrestis*), indicating ancient diversification events within basal eudicots (Group et al., 2016; Zhou et al., 2018).

## 5 Conclusion

This comprehensive study underscores the considerable interspecific and regional variation in secondary metabolite composition among *Papaver* species, reflecting the complex interplay between genetic factors and environmental conditions. Among the species and regions examined in this study, *P. hybridum* appeared comparatively promising, as it exhibited higher levels of total phenolic and flavonoid compounds, stronger antioxidant activity, and elevated concentrations of codeine, particularly in samples from Khuzestan and Kermanshah. These findings suggest potential pharmacological relevance that merits further investigation. In contrast, *P. rhoeas* generally showed lower levels of the assessed bioactive compounds under the specific conditions and sampling areas considered here, indicating that its medicinal applications could be more limited; however, additional research across a broader range of environments and populations is necessary to confirm these observations and fully assess their significance.

The strong positive correlations between total phenolics, flavonoids, and antioxidant activities (DPPH and FRAP assays) confirm the central role of polyphenols in oxidative stress mitigation. The distinct clustering of alkaloids such as codeine and papaverine in PCA further suggests that these compounds are regulated by different metabolic pathways, largely independent from those governing antioxidant activity. Overall, the PCA showed no clear separation among provinces, indicating broadly similar phytochemical and antioxidant profiles across regions. Furthermore, our Mantel test suggests that genetic differences only partly explain the biochemical variation among populations, with environmental factors likely playing a major role. Although ITS sequencing provided useful genetic information, it did not strongly predict phytochemical profiles. Therefore, DNA barcoding should be combined with chemical analyses to better characterize diversity and guide selection for medicinal or ecological uses.

Finally, this research not only enriches our understanding of the phytochemical and genetic diversity within the *Papaver* genus but also provides a solid foundation for the targeted selection of species and regions for pharmaceutical, nutraceutical, and ecological applications.

## Data Availability

The raw data supporting the conclusions of this article will be made available by the authors, without undue reservation, to any qualified researcher.
